# Implementation of the WHO Approved “Tailoring Antimicrobial Resistance Programs (TAP)” Reduces Patients’ Request for Antibiotics

**DOI:** 10.3390/antibiotics9080507

**Published:** 2020-08-12

**Authors:** Nasser M. Kaplan, Yousef S. Khader, Mahmoud A. Alfaqih, Rami Saadeh, Lora Al Sawalha

**Affiliations:** 1 Department of Pathology and Microbiology, Faculty of Medicine, Jordan University of Science and Technology, 22110 Irbid, Jordan; nmkaplan@just.edu.jo; 2 Department of Community Medicine and Public Health, Faculty of Medicine, Jordan University of Science and Technology, 22110 Irbid, Jordan; yskhader@just.edu.jo (Y.S.K.); rasaadeh@just.edu.jo (R.S.); 3 Department of Physiology and Biochemistry, Faculty of Medicine, Jordan University of Science and Technology, 22110 Irbid, Jordan; 4 Anti-Microbial-Resistance Officer, World Health Organization, Jordan Country Office, 11181 Amman, Jordan; alsawalhal@who.int

**Keywords:** antibiotics, microbial resistance, upper respiratory tract infections

## Abstract

The misuse of antibiotics is a worldwide public health concern. Behavioral Intervention programs that aim to reduce patients’ own request for antibiotics during their visit to primary care clinics is an attractive strategy to combat this problem. We tested the effectiveness of a behavioral modification method known as the Tailoring Antimicrobial resistance Programs (TAP) in reducing the request for antibiotics by patients visiting primary care clinics for mild upper respiratory tract infections (URTIs). A stratified cluster randomized design with two groups pre-post, comparing intervention with the control, was conducted in six health centers. TAP was implemented for eight weeks. Request for antibiotics was assessed before (period 1) and after introducing TAP (period 2). The percentage of patients or their escorts who requested antibiotics in period 1 was 59.7% in the control group and 60.2% in the intervention group. The percentage of patients who requested antibiotics did not significantly change between period 1 and 2 in the control group, who continued to receive the standard of care. The above percentage significantly decreased in the intervention group from 60.2% to 38.5% (*p* < 0.05). We conclude that behavioral change programs including TAP are a viable alternative strategy to address antibiotic misuse in Jordan.

## 1. Introduction

Antimicrobial resistance is a threat to the public health sector worldwide [1]. Many factors contribute to this problem; however, the misuse and/or overuse of antibiotics are established as the major driving forces [2,3]. Indeed, it was estimated that up to 50% of all antimicrobials globally prescribed to patients are not even necessary [4,5]. It is interesting to note that most of the unnecessary antibiotic prescription takes place in the primary care setting [6], with the biggest percentage of unnecessary antibiotics being prescribed for patients with upper respiratory tract infections (URTIs) [6].

Jordan is a developing country in the Middle East and North Africa (MENA) region. The misuse of antibiotics by consumers, including the use of antibiotics without prescriptions, was widely documented in Jordan and in the region [7,8,9,10,11,12]. Other forms of antibiotic misuse in the MENA region include the use of antibiotics for improper indications, including to fight viral infections [13,14,15,16].

Despite the magnitude of the antibiotic misuse problem, most of the countries in the MENA region have no laws and/or legislations that prohibit dispensing antibiotics without a proper prescription [8,17]. Moreover, countries that have relevant legislation in place do not have proper surveillance systems and/or do not adequately enforce relevant laws [18]. Interestingly, knowledge about antibiotic misuse and antimicrobial resistance by itself, without being coupled with behavioral change interventions, does not seem to be an efficient strategy to enforce better antibiotic stewardship [19,20,21]. This observation might be explained by the complexity of the factors that affect antibiotic misuse which appears to be influenced by a plethora of cultural and social factors [19,21]. For example, several reports demonstrated that the specialty of the health care provider, patient education and other patient socio-economic factors guide the antibiotic prescription patterns of physicians [22,23,24,25].

Tailoring Antimicrobial Resistance Programs (TAP) is a behavioral change methodology developed by the World Health Organization (WHO) Eastern Mediterranean Regional Office (EMRO) to modify the behaviors that drive antimicrobial resistance (AMR). TAP methodology not only aims to identify barriers against proper behavior but also identifies the incentives that drive such a behavior. TAP proposes guidelines for (a) the design of proper behavioral change strategies, (b) implementation of such strategies and (c) evaluation of the results of any behavioral intervention. The Ministry of Health in Jordan joined the WHO TAP in November 2018 to pilot a behavioral change intervention that aims to reduce the prescription of antibiotics for viral URTIs in a primary healthcare setting. This study presents and discusses the findings of the TAP intervention, specifically its effect in reducing the percentage of patients that request antibiotics. Additionally, the study investigated the association of several socioeconomic factors with changes in antibiotic request by the patients following TAP intervention.

## 2. Results

### 2.1. The Characteristics of the Study Subjects

**Figure 1:**
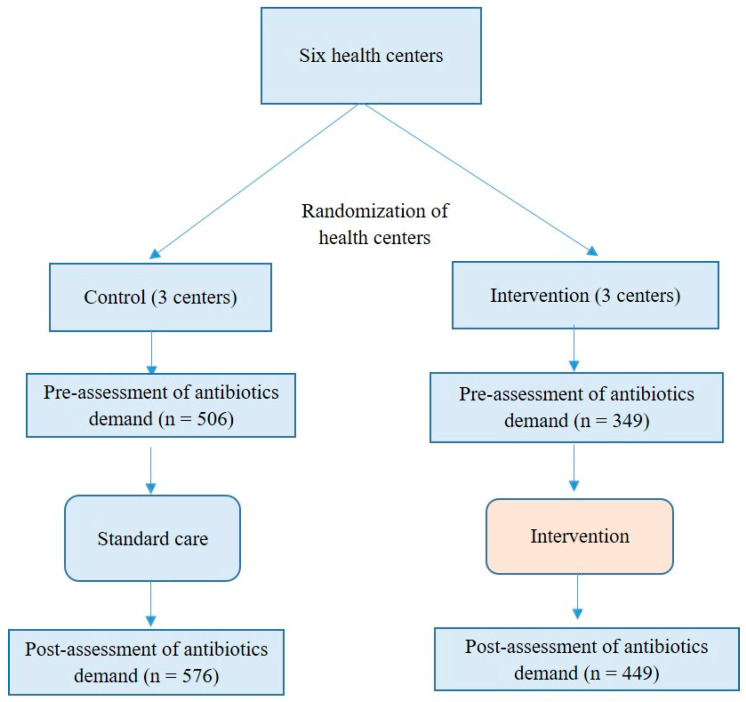
A flow chart that explains the design of the study.

A total of 855 subjects (506 in the control group and 349 in the intervention group) participated in the study in period 1 before the implementation of the intervention. In period 2, following the intervention, a total of 1025 subjects (576 in the control group and 449 in the intervention group) were enrolled in the study (Figure 1). 

A stratified cluster randomized trial with two groups pre-post design, comparing intervention with the control (standard care), was used in the study. The study was performed in six health centers in Amman. The centers were randomized into two groups (three centers each). In period 1, there was a pre-assessment of antibiotic request. In period 2, following application of the intervention or maintenance of standard treatment care, there was a re-evaluation of antibiotic request among enrolled patients.

The socio-demographic characteristics of the subjects of the control and intervention groups in periods 1 and 2 are shown in Table 1. In period 1, 17.8% of the subjects in the control group and 7.2% of the subjects in the intervention group were children (*p* < 0.001). In period 2, almost one quarter of the subjects in both groups were children (*p* = 0.320). In period 1, subjects of the intervention group were significantly younger. Moreover, a significantly higher number of the above subjects did not hold a university degree. More than half of the subjects (55.9%) in the control group and 33% of the subjects in the intervention group were new patients. In period 2, a significantly lower number of subjects in the intervention group received college/university education than subjects in the intervention group (*p* < 0.001). The characteristics of the subjects significantly differed between period 1 and period 2 in both control and intervention groups.

### 2.2. Effect of TAP Intervention on Antibiotics Request among Study Subjects

**Figure 2:**
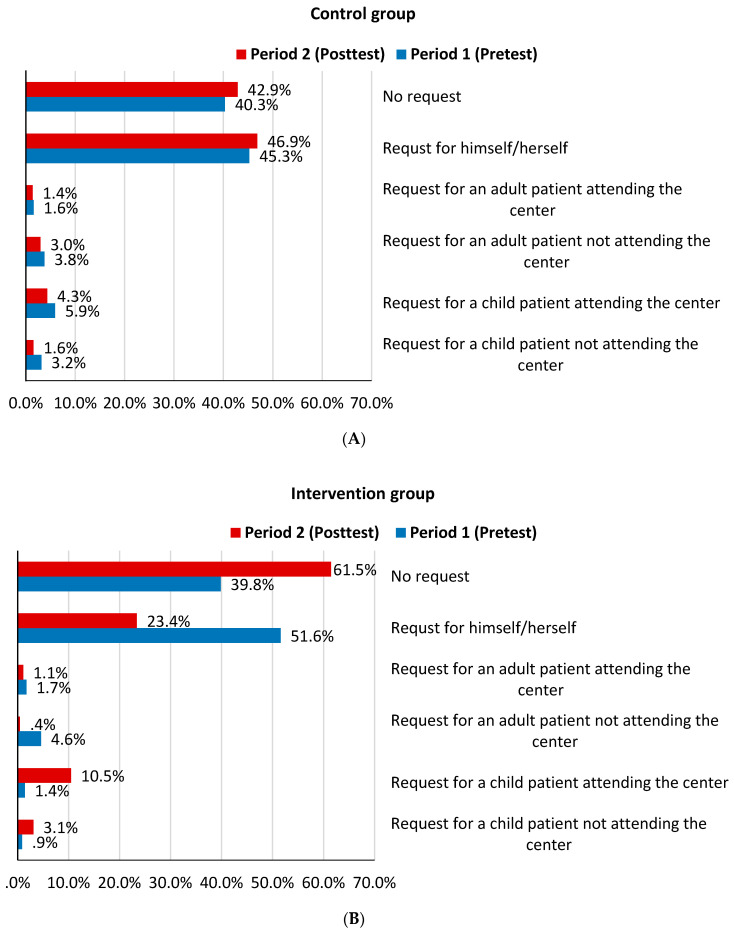
The pattern of antibiotics requests in the control or intervention groups. A horizontal bar graph displaying the pattern of antibiotics request in period 1 (**blue**) and period 2 (**red**) in (**A**) Control or (**B**) Intervention groups. Each bar represents the percentage of individuals that either did not request the antibiotics, requested the antibiotics from themselves, requested the antibiotics for an adult patient attending the center, requested the antibiotics for an adult patient not attending the center, requested the antibiotics for a child patient attending the center or requested the antibiotics for a child patient not attending the center.

The percentage of patients or their escorts who requested antibiotics in period 1, before the implementation of the intervention, was 59.7% in the control group and 60.2% in the intervention group (*p* = 0.886) (Figure 2). While the percentage of requesting antibiotics did not change significantly between period 1 and 2 in the control group (*p* = 0.393), this percentage decreased significantly in the intervention group from 60.2% to 38.5% (*p* < 0.05) (expressed as no request in Figure 2). The relative percent of reduction in the percentage of subjects who requested antibiotics between the two periods in the intervention group was 36% (absolute difference of 21.7%).

### 2.3. Pattern of Antibiotics Request

In the intervention group, the percentage of patients or their escorts who requested antibiotics for themselves decreased from 51.6% before the intervention to 23.4% following the intervention, with a relative percent of reduction of 45.7% (Figure 2). In the control group, the above described percentage did not change significantly between period 1 and period 2 (*p* = 0.359). About 45.3% and 46.9% of participants requested antibiotics for themselves in period 1 and period 2, respectively.

### 2.4. Reasons for Requesting Antibiotics

Overall, among those who requested antibiotics in both the control and intervention groups (n = 1014), the most common reasons were sore throat (36.7%) followed by cough (27.5%). The health-related complaints that motivated subjects of this study to request antibiotics differed between the control and intervention groups (Table 2). In the control group, the most common reasons for requesting antibiotics were sore throat, followed by flu, and then cough (Table 2). In the intervention group, the main complaints were sore throat, followed by pain upon swallowing, fever, and then cough (Table 2).

### 2.5. Type of Antibiotics Requested 

Of those who requested antibiotics, 961 (94.8%) requested a specific type of antibiotics (data not shown). It is interesting to note that more than three quarters (78.1%) requested amoxicillin/clavulanic acid (Amoclan) (data not shown).

### 2.6. Factors that Influence Antibiotics Request

Table 3 shows a multivariate analysis of the effect of multiple factors on the decision of the study subjects to request antibiotics from the prescriber (i.e., physician). In the control group, the variables that were significantly associated with requesting antibiotics by the patients were age of the patient, type of patient (regular vs. first appointment), level of education of the patient and the specialty of the health care provider. Antibiotics were more likely to be requested by, or for, adult patients compared to patients who were children (Odds Ratio (OR) = 1.7) (Table 3). Regular patients were more likely to request antibiotics compared to patients visiting that specific physician for the first time (OR = 2.5) (Table 3). Patients with no formal education, primary education, secondary education or with professional training were more likely to request antibiotics than patients with college/university education. Patients who were visiting a family doctor were less likely to request antibiotics than patients visiting general practitioners (OR =0.5). On the other hand, in the intervention group, our analysis showed that antibiotic request by subjects was significantly lower following the implementation of the intervention (OR = 0.4). The only other variable in the intervention group to significantly affect antibiotic request was primary or secondary education compared to having no formal education. Our results showed that having primary or secondary education significantly increased the odds of requesting antibiotics.

## 3. Discussion

The misuse of antibiotics in primary care is a major contributor to antibiotic resistance [5]. URTIs are common presentations seen in the general practice [26]. URTI without complications is most often caused by a virus [27]. Antibiotics have no efficacy in the treatment of viral infections, but are nevertheless often prescribed for their treatment [28,29].

In this study, using a stratified cluster randomized trial with two groups pre-post design, we evaluated the effect of the TAP intervention in reducing the percentage of patients that request antibiotics. The above design allowed for a comparison of the intervention group receiving TAP with a control group in which standard care was maintained. In addition to collecting information on the percentage of patients who requested antibiotics, the research team collected data of several factors previously reported to affect antibiotic vigilance such as gender, age, level of education of the patient and the specialty of the health care provider. The study design also differentiated between patients visiting the physician for the first time and returning patients. Recruitment to the study was restricted to patients complaining from URTIs. 

In this pilot study conducted on patients visiting six health centers in the capital city of Jordan, Amman, we were the first group to demonstrate the efficacy of the TAP program in reducing the percentage of patients that request antibiotics from their health care provider in a primary care setting. Notably, our findings also indicated that the TAP program achieved its goal independent of all the other variables that might influence antibiotic requests by the patients. 

In the absence of any intervention (in our case the TAP program) our findings indicated that more than half of the patients diagnosed with mild URTIs would request antibiotics for the treatment of their illness. This result is analogous with other reports which suggested that patient pressure or “perceived pressure” is a major driver of the lack of antibiotic vigilance [30,31]. The above figure, showing that most patients request antibiotics from their primary health care provider as a result of illnesses that do not normally require antibiotics, may reflect the lack of public awareness programs on the harmful effects of the unnecessary use of antibiotics. Although this observation is alarming from a public health standpoint, the fact that the TAP method successfully reduced the number of patients that request antibiotics shows that positive behavioral change could be achieved in the patient population and invites the application of the TAP method on a larger scale.

An interesting finding of this investigation was the difference observed in the percentage of patients that request antibiotics based on the specialty of the health care provider. For example, in the control group, it was observed that patients were more likely to request antibiotics from general practitioners vs. family medicine specialists. In Jordan, general practitioners start their appointment following one year of vocational training only (internship), without enrollment in any residency program. On the other hand, to become a family medicine specialist in Jordan, candidates must finish their vocational training, enroll in a structured residency program and pass a national board exam. The exact reason behind the above disparity in antibiotic request between patients seen by different specialists is unknown but could be related to family medicine specialists building better communication and assertive skills during their residency programs [32]. If the above explanation turns out to be partially responsible for this disparity, a solution for this problem would be to offer general practitioners Continuing Medical Education (CME) courses in communication skills and antibiotic stewardship. These courses would help mend the gap created by a longer study path to become a family medicine specialist. 

This investigation has a few limitations. First, this study was conducted in Amman, the capital city of Jordan. Although the findings of this study are very promising, the adoption of the TAP program as a method to achieve better antibiotic vigilance requires testing the program across different geographic regions. For example, the level of education, a variable shown in this study to affect antibiotic stewardship, might be different in Amman from other geographic regions in the country. Second, the research team failed to collect information on the volume of patients examined by physicians on a single day in the clinic. This variable was shown in several reports to affect consultation time with the patient, and was significantly associated with an excess, often unnecessary, antibiotic prescription [33,34]. Indeed, it would be interesting to evaluate if implementation of the TAP actually increased the consultation time with the patients and how that affected the overall revenue of the medical practice/clinic. Despite these limitations, this study is the first in Jordan and should be informative to public health policy makers and health care workers interested in antibiotic stewardship with regards to the size of the antibiotic misuse problem and the feasibility of reducing this problem with a simple behavioral approach.

## 4. Materials and Methods

### 4.1. Study Design, Site Selection and Randomization

A stratified cluster randomized trial with two groups pre-post design, comparing intervention with the control (standard care), was conducted in the period between August and November of 2019. The standardized behavior change intervention was implemented for eight weeks in the intervention group. The demand for antibiotics was assessed among patients with mild URTIs attending the intervention and control centers before and after introducing the behavior change intervention. Written consent was requested from all patients before the interviews. No identifiers were collected. Approval from the Jordan Ministry of Health Ethical Review Board was obtained prior to conducting the study. All interviews were conducted in a closed room to ensure privacy and confidentiality.

Six health centers in Amman, Jordan were selected using a stratified cluster randomized sampling strategy. The centers were classified into three strata—small, medium and large—based on the number of physicians and monthly patient visits obtained from statistics of the year 2018. Out of each stratum, one center was randomized to the intervention group and another center to the control group, resulting in three centers in each group.

### 4.2. Patient Recruitment 

General practitioners, family medicine specialists, pediatricians and internal medicine specialists were trained to interview patients attending the clinic for mild URTIs or to obtain medications for relatives with mild URTIs. The practitioners used a semi-structured questionnaire before and after the intervention to assess the demand for antibiotics. The questionnaire was pilot tested on 30 patients and revised accordingly. All consecutive patients of all ages diagnosed with URTIs who visited the selected health centers during the working hours for the duration of the study period were included. Only patients visiting general practitioners, family medicine specialists, pediatricians, and internal medicine specialists were included. Patients diagnosed with infections other than URTIs were excluded.

### 4.3. Intervention

A strategic behavior change intervention package was designed and implemented in the three intervention centers. As part of the intervention, physicians were trained, by a WHO expert in the area of antimicrobial resistance and a consultant in communication, to adopt a more proper behavior relevant to antibiotic prescription and to communicate with patients who insist on receiving antibiotics for viral URTIs. A 1-day training workshop was held in the premises of the Ministry of Health (MoH). During the training, physicians were trained on the current national guidelines for prescribing antibiotics to patients with URTIs and were trained on the best approaches to manage discussions with difficult patients. The physicians in the intervention centers received a copy of the clinical guidelines for the diagnosis and treatment of URTIs and were instructed to adopt the guidelines in their practices. A commitment was obtained from prescribers to become advocates for the proper use of antibiotics for URTIs and to join the intervention by signing a commitment board. 

Posters were placed in the waiting areas to advise patients not to request antibiotics from their doctors and to always consult a doctor before antibiotics’ administration, and leaflets about the proper use of antibiotics were distributed to patients. During the routine patient consultation in the intervention centers, physicians requested each patient to answer a quiz about the indication and proper use of antibiotics. Then, the physician held a short discussion (2–3 min) about the answers to the questions, encouraged patients to reduce their requests for antibiotics, and provided patients with information about the antibiotics and the consequences of improper prescription. Peer-to-peer weekly coffee sessions were held and moderated by the MoH staff to strengthen the bonds between colleagues and managers as a single entity that reduced the unnecessary use of antibiotics. Moreover, the strategies used to implement the behavior change, including the roles of the prescribers and patients, are shown in Table 4.

The MoH staff coordinating the project regularly visited the clinics during the intervention, observed the physicians’ practices, and filled out the monitoring forms. The monitoring form included information on the physicians’ adherence to the study protocol and the number of patients treated.

### 4.4. Sample Size

A minimum sample size needed to assess the effect of the intervention on the change in the percentage of patients who request antibiotics for URTIs in a pretest-posttest nonequivalent control group design was calculated using G*Power. Assuming that the percentage of patients who request antibiotics for URTIs in the selected health centers is 50%, the sample size needed to detect a change of 12% in this percentage following the intervention (at a level of significance of 0.05 and a power of 80%) is 370 patients in the intervention group (370 at pretest and 370 at posttest) and 370 patients in the control group (370 at pretest and 370 at posttest). This is the minimum sample size with enough power to determine the impact of the intervention, taking into consideration that the analysis will be stratified by demographic and clinical characteristics.

### 4.5. Statistical Analysis

Data were analyzed using IBM SPSS, version 20 (IBM Corp., Armonk, NY, USA). Data were described using means, standard deviations, and percentages. Chi-square test was used to compare the percentage of patients who requested antibiotics for URTIs between intervention and control groups and between the two periods within each group. The same test was used to compare demographic and other categorical variables between intervention and control groups and between the two periods within each group. Binary logistic regression was used to test for the change in request for antibiotics over time, after adjusting for patients’ characteristics. The interaction term between period (pretest (period 1)/ posttest (period 2)) and group (intervention/control) was tested. A *p*-value of less than 0.05 was considered statistically significant.

## 5. Conclusions

In conclusion, in this pilot study evaluating the TAP program as a measure to achieve proper antibiotic stewardship in Jordan, we provide evidence on its efficacy, simplicity and feasibility. Given the small scale of this investigation, we recommend testing the program on a larger scale and across multiple health sectors in the country. We anticipate that the interventional program described in this investigation might be adopted as a public health method to address the misuse of antibiotics in Jordan.

## Figures and Tables

**Table 1:** The socio-demographic characteristics of patients in the control and intervention groups during period 1 and period 2.

	Period 1 (Pretest)					Period 2 (Posttest)				
Variable	Control Group		Intervention Group		*p*-value	Control Group		Intervention Group		*p*-value
	n	%	n	%		n	%	n	%	
**Gender**					0.666					0.488
**Male**	297	58.7	210	60.2		369	64.1	297	66.1	
**Female**	209	41.3	139	39.8		207	35.9	152	33.9	
**Age**					<0.001					0.320
**Children (<18 year)**	90	17.8	25	7.2		142	24.7	123	27.4	
**Adults (≥18 year)**	416	82.2	324	92.8		434	75.3	326	72.6	
**Nationality**										
**Jordanian**	475	93.9	332	95.1		558	96.9	427	95.1	
**Non-Jordanian**	31	6.1	17	4.9		18	3.1	22	4.9	
**Education**					<0.001					<0.001
**No formal education**	72	14.2	43	12.3		68	11.8	60	13.4	
**Primary education**	84	16.6	98	28.1		106	18.4	141	31.4	
**Secondary education**	155	30.6	114	32.7		161	28	151	33.6	
**Professional training**	35	6.9	16	4.6		20	3.5	3	0.7	
**College/University education**	160	31.6	78	22.3		221	38.4	94	20.9	
**Marital status**					<0.001					0.158
**Married**	332	65.6	265	75.9		359	62.3	284	63.3	
**Single**	60	11.9	30	8.6		43	7.5	29	6.5	
**Divorced/Widow**	24	4.7	29	8.3		32	5.6	13	2.9	
**Children**	90	17.8	25	7.2		142	24.7	123	27.4	
**Patient’s type**					<0.001					0.606
**New**	283	55.9	115	33		298	51.7	225	50.1	
**Regular**	223	44.1	234	67		278	48.3	224	49.9	

**Table 2:** The most frequent patient complaints associated with requesting antibiotics in the control and intervention groups in period 1 or 2.

Complaint	Control				Intervention			
	Period 1 (Pretest)		Period 2 (Posttest)		Period 1 (Pretest)		Period 2 (Posttest)	
	n	%	n	%	n	%	n	%
**Sore Throat**	104	34.4	118	35.9	103	49.0	56	32.4
**Flu**	67	22.2	64	19.5	33	15.7	32	18.5
**Cough**	84	27.8	119	36.2	34	16.2	42	24.3
**Pain on swallowing**	39	12.9	50	15.3	40	19.0	42	24.3
**Cold**	31	10.3	21	6.4	8	3.8	27	15.6
**Influenza**	61	20.2	62	18.8	27	12.9	40	23.1
**Fever**	58	19.2	50	15.2	16	7.6	49	28.3
**Nasal Congestion**	42	13.9	30	9.1	18	8.6	14	8.1
**Breathing Difficulties**	33	10.9	59	17.9	11	5.2	12	6.9
**Nasal secretion**	34	11.3	24	7.3	11	5.3	9	5.2
**Sneezing**	27	8.9	22	6.7	5	2.4	7	4.0
**Weakness**	16	5.3	14	4.3	14	6.7	8	4.6

**Table 3:** Multivariate analysis of factors associated with antibiotics demand in the control and intervention groups.

Variable	Control Group				Intervention Group			
	OR	95% Confidence Interval		*p*-Value	OR	95% Confidence Interval		*p*-Value
**Time (post vs. pre)**	1.0	0.8	1.4	0.713	0.4	0.3	0.6	<0.001
**Specialty of health care provider**								
**General practitioner**	1				1			
**Family Medicine**	0.5	0.4	0.7	<0.001	1.2	0.8	1.6	0.366
**Pediatrics**	1.1	0.8	1.7	0.519	1.0	0.6	1.6	0.927
**Internal Medicine**	0.9	0.5	1.7	0.792				
**Education**								
**No formal education**	2.9	1.9	4.5	<0.001	1.0	0.6	1.7	0.968
**Primary education**	2.3	1.6	3.3	<0.001	1.8	1.1	2.7	0.010
**Secondary education**	1.7	1.3	2.4	0.001	1.9	1.3	2.8	0.002
**Professional training**	5.7	2.8	11.6	<0.001	0.8	0.3	2.3	0.74
**College/university education**	1							
**Age of patient (adults vs. children)**	1.7	1.1	2.5	0.006	1.3	0.8	2.1	0.229
**Type of patient (regular vs. new)**	2.5	1.9	3.3	<0.001	1.2	0.9	1.7	0.153

**Table 4:** Behavioral change strategies for prescribers or patients.

		Behavioral Barrier	Behavioral Domain	Intervention Function		Intervention		Activities	
**Behavioral change strategies and activities for prescribers**									
**1**		Limited communication skills to manage patient pressure of antibiotics for viral infections	Skills	Physical capability		To improve counselling and negotiation skills of doctor to better manage patient demand for antibiotics for viral infections		A training workshop for communication skills	
**2**		Limited knowledge of guidelines and alternative treatments for viral infections	Knowledge	Psychological capability		To increase doctors’ knowledge of guidelines and alternatives to antibiotics		A prescriber reference booklet including national guidelines for viral Upper Respiratory Tract Infections (URTIs)	
**3**		Social norms: patient culture of demanding antibiotics and expecting to best know the suitable treatment for self and family.	Social	Social opportunity		To emphasize the professional role of doctors as the best one to diagnose illness and prescribe antibiotics		A conversation/quiz with patients Commitment board	
**4**		Peer pressure to prescribe antibiotics for viral infections	Professional role	Reflective motivation		To strengthen the bonds between colleagues and managers as one entity that reduced unnecessary use of antibiotics		Peer to peer weekly coffee session	
**Behavioral change strategies and activities for patients**									
**1**	Limited knowledge of proper use of antibiotics and Antimicrobial Resistance (AMR)		Knowledge		Psychological capability		To raise knowledge about antibiotics and AMR		A quiz during patient consultation
**2**	Limited knowledge that antibiotics are not a solution for viral infections		Knowledge				To raise awareness about alternative therapies		A quiz during patient consultation
**3**	Limited understanding of the consequences of improper use of antibiotics		Belief in consequences		Reflective motivation		To label families who do not consume antibiotics for viral infections as healthy and wealthy families		A quiz during patient consultation Poster
**4**	Social norms linked with beliefs that people know which antibiotics work best for them		Social		Social environment		To emphasize doctors’ role as the best to diagnose patients following the Arabic proverb “give the bread to the baker”.		A quiz during patient consultation **Poster**: Never demand antibiotics from your doctor. Always consult your doctor before taking antibiotics
**5**	No plans to change behavior		Intentions/goals				To encourage change in social norms by using people who do not use antibiotics as a reference group		Commitment board

## Abbreviations

**Abbreviation**	**Full Term**
TAP	Tailoring Antimicrobial resistance Program
URTIs	Upper Respiratory Tract Infections
MENA	Middle East and North Africa
WHO	World Health Organization
EMRO	Eastern Mediterranean Regional Office
AMR	Antimicrobial resistance
SPSS	Statistical Package for the Social Sciences
OR	Odds Ratio
CME	Continuing Medical Education
